# Post-operative management of children after lung transplantation

**DOI:** 10.1016/j.jhlto.2025.100301

**Published:** 2025-05-27

**Authors:** Nicolaus Schwerk, Julia Carlens, Harald Köditz, Fabio Ius, Nicholas Avdimiretz, Don Hayes, Melinda Solomon

**Affiliations:** a Clinic for Pediatric Pneumology, Allergology and Neonatology, Hannover Medical School, Hannover, Germany; b German Center for Lung Research (DZL/BREATH), Hannover, Germany; c Clinic for Pediatric Cardiology and Intensive Care Medicine, Hannover Medical School, Hannover Medical School, Hannover, Germany; d Department of Cardiothoracic, Transplant and Vascular Surgery, Hannover Medical School, Hannover, Germany; e Division of Respiratory Medicine, British Columbia Children’s Hospital, University of British Columbia, Vancouver, Canada; f Heart Institute and Division of Pulmonary Medicine, Cincinnati Children’s Hospital and Medical Center, University of Cincinnati College of Medicine, Cincinnati, OH; g Division of Respiratory Medicine, The Hospital for Sick Children, University of Toronto, Toronto, Canada

**Keywords:** Lung transplantation, Children, Adolescents, Post-operative management, Complications, Intensive care

## Abstract

Post-operative care for children and adolescents who undergo lung transplantation is a challenge because of the potential for numerous complications during this period, which can considerably impact the short- and long-term outcomes. The immediate post-operative phase is particularly critical, and complications are frequent; therefore, knowledge, early recognition, and appropriate treatment of these complications are imperative and can only be achieved through close collaboration between a wide range of medical specialties. The aim of this review is to provide an abbreviated overview of the optimal post-operative management of children in an intensive care unit, as well as to describe frequently occurring complications and their treatment.

## Background

Lung transplantation (Ltx) is a treatment option for children and adolescents with end-stage lung disease who are deemed candidates. Although outcomes remain inferior to other solid organ transplants, improvements in bridging strategies, organ preservation, surgical techniques, and post-operative management have resulted in decreased mortality over the past two decades, with an actual median survival of 5.7 years.^1^ Nevertheless, the mortality rate during the first post-Ltx year remains high, ranging between 15% and 20%, with most deaths occurring during the first three post-operative months.^2^ Notably, complicated post-operative courses have a negative impact on long-term survival and quality of life, for example, due to immobility, chronic infection, and/or ventilator-associated lung injury and subsequent chronic lung allograft dysfunction or renal impairment, including the need for dialysis.^3^, ^4^, ^5^, ^6^, ^7^ Optimal post-operative management immediately after Ltx is critical for successful long-term outcomes and varies considerably depending on the underlying diagnosis, such as the critically ill infant with surfactant metabolism disorder and secondary pulmonary hypertension (PH) or an adolescent with cystic fibrosis.^8^, ^9^ The purpose of this review is to provide a brief overview of the optimal post-operative management of children in the intensive care unit and to describe common complications and their management. Immunosuppressive therapy and the diagnosis and treatment of acute rejection are not discussed here, as they are described in detail elsewhere in this issue.

### Influence of the underlying disease and comorbidities on early post-operative outcome

The influence of underlying disease, age, and the presence of comorbidities on early post-operative outcome and long-term survival is significant and must be considered during the evaluation process.^10^ When listing patients for Ltx, it is essential to define individualized treatment plans that consider these factors. Whereas cystic fibrosis (CF) was the most common indication until recently,^1^ diffuse parenchymal lung disease and PH are now the most common indications.^11^, ^12^ Specific indications and how these are shifting are described in an accompanying article in this special issue. Beyond these evolving issues for pediatric Ltx indications, post-operative outcomes are adversely affected by other factors such as impaired renal function, invasive ventilation prior to transplantation, and single Ltx.^2^ The most common causes of death in the first months after Ltx are primary graft failure, infection, cardiovascular complications, and multiple organ failure.^1^, ^8^, ^9^, ^13^, ^14^

### Monitoring

Monitoring is essential, as the optimization of patient's hemodynamic, ventilation, temperature, nutrition, and metabolism is the key to improving patients' survival. It includes invasive blood pressure measurement, electrocardiography, oxygen saturation assessment, ventilator setting controls, and strict monitoring of fluid balance. For optimal hemodynamic management, monitoring of central venous pressure should be considered. Therefore, in addition to standard non-invasive and invasive monitoring, many centers use a Swan-Ganz catheter to assess pulmonary artery pressure, wedge pressure, and cardiac index.^8^, ^9^, ^15^, ^16^ In patients undergoing extracorporeal membrane oxygen (ECMO) support, care might even be more complex as the patient is at risk of circuit related complications (ie, bleeding complications, cannula dislocation, rhabdomyolysis, and compartment syndrome) and impaired perfusion of the leg distal to the cannula.^17^ Serial Chest radiographs are necessary to detect opacities, atelectasis, edema, effusions, or hemorrhage. Regular echocardiographic monitoring is important in patients with PH or cardiac dysfunction to monitor right and left ventricular function and to rule out anastomotic stenosis of the pulmonary vessels and thrombosis. Frequent blood sampling is essential to determine blood counts, inflammatory parameters, electrolytes, blood gases, cardiac and hepatic enzymes, ammonia, amylase, lipase, renal function, and plasma levels of immunosuppressants. In addition, donor-specific antibodies should be monitored based on the clinical course of the patient.^18^

### Bronchoscopy

**Figure 1:**
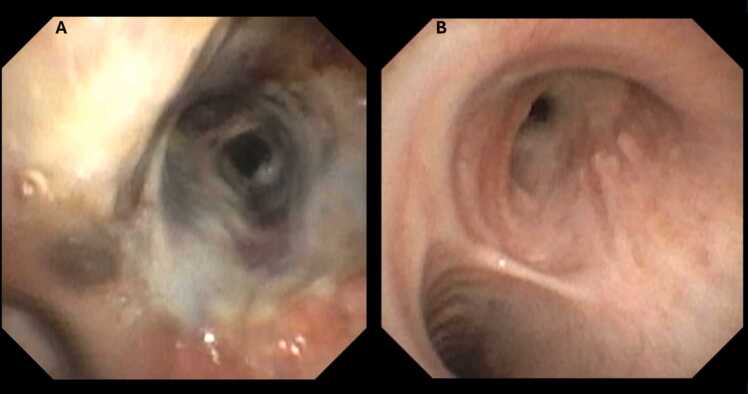
Bronchoscopic view of the right main bronchus in a patient with CF after lung transplantation; 5 days after transplantation with severe desquamative bronchitis and detection of Pseudomonas aeruginosa **(A)** and 8 weeks after transplantation with a resulting severe stenosis distal to the anastomosis in the intermediate bronchus **(B)**.

Bronchoscopy plays a crucial role in the early post-operative period. It helps to detect potential complications such as anastomotic dehiscence, stenosis, hemorrhage, secretion accumulation, and infection (Figure 1). During surgery, anastomotic stenosis or insufficiency should be excluded while blood, secretions, and cellular debris should be removed from the airway. Before extubation, regular bronchoscopic surveillance should be performed to exclude anastomotic and airway complications and to obtain material for microbiological and virological testing. The benefit of early bronchoalveolar cytology in the early post-operative period is uncertain because neutrophilic inflammation is typically predominant and Sudan-positive alveolar macrophages can be detected even without aspiration events. The frequency of bronchoscopy after extubation depends on the clinical condition of the patient and the radiological findings.^19^, ^20^, ^21^, ^22^

Although not proven by systematic studies, care should be taken to use as little suction as possible to avoid causing airway edema. Allograft rejection occurs frequently in post-operative period, so monitoring and management in pediatric Ltx recipients are discussed in an accompanying article in this special issue.

### Sedation

The primary objective of sedation is to enhance the patient's well-being, alleviate anxiety and stress, and avert desynchronization during ventilation.^15^ The intensity of sedation and the selection of medication must be determined on an individual basis, considering the patient's age and neurocognitive development.^8^ The overarching aim should be to minimize the use of sedatives and to employ non-pharmacological interventions to calm an anxious or agitated child.^15^ Caregivers play a pivotal role in this regard; therefore, they should be permitted to remain with their child. In addition, the contributions of nursing staff, behavioralists, and child life specialists, if available, are of great importance. The post-operative administration of benzodiazepines is a common practice; however, prolonged use is associated with severe adverse effects, including delirium, drug withdrawal symptoms, and immobilization.^23^ Alternatives with a more favorable risk-benefit profile include alpha-2 antagonists like dexmedetomidine.^24^ Other commonly used sedatives include propofol and ketamine. Propofol, when administered over an extended period, carries a risk of life-threatening complications, including propofol-related infusion syndrome, characterized by rhabdomyolysis, severe lactic acidosis, and subsequent multiple organ failure.^25^

### Pain management

Optimal pain management is essential to protect children from stress and psychological trauma while allowing extubation and mobilization as early as possible.^8^, ^9^, ^17^ In many adult and a few pediatric centers, regional anesthetic techniques such as thoracic epidural anesthesia have become established procedures. This approach allows for early extubation, mobilization, and reduction in opiates.^26^, ^27^, ^28^ Patient-controlled analgesia with opiates is another alternative approach.^29^ When opiates are used, special attention must be paid to gastrointestinal complications. Acetaminophen or metamizole are other treatment options that can reduce the need for opiates, but they have associated side effects, and, in the United States, metamizole is not Food and Drug Administration approved due to the risk of agranulocytosis. Furthermore, for metamizole, thrombocytopenia, cytopenia, and hypotensive circulatory reactions should be noted, and for acetaminophen, hepatotoxicity and hepatic failure. In addition, non-pharmacologic interventions as already mentioned in the section *sedation,* are of great importance. Indication for pain management must always be carefully evaluated, with administration kept to a necessary minimum.^8^, ^9^, ^15^ Non-steroidal anti-inflammatory drugs should be avoided due to their potential for nephrotoxicity.

### Ventilation

There are experimental data which show, that Ltx recipients are at particularly high risk to develop ventilator-induced lung injury.^30^, ^31^ In this context, lung-protective ventilation strategies are essential during the post-operative period.^32^, ^33^ Due to the lack of evidence on the optimal ventilation strategy after Ltx, the concepts of acute respiratory distress syndrome ventilation with permissive hypercapnia are often applied, as the benefits of lung-protective mechanical ventilation (MV) have been shown to improve outcomes.^34^, ^35^ Excessive ventilation pressures have an additional deleterious effect on the microcirculation, and as anastomotic sites are subject to limited blood supply, this could lead to airway complications. Therefore, ventilation strategies are aimed at achieving tidal volumes of 5-6 ml/kg with avoidance of peak inspiratory pressures > 26 cmH_2_O and positive end-expiratory pressure >8 cm H_2_O. Hypoxemia induces vasoconstriction, which can lead to impaired lung perfusion and increased pressure in the pulmonary arteries. Consequently, it is pivotal to ensure that oxyhemoglobin saturation levels are maintained at an adequate level. On the other hand, to counteract the potential for further inflammation caused by free radicals, it is advisable to maintain the fractional inspiratory oxygen concentration (FiO_2_) at a minimum to maintain adequate saturation levels.^36^ Oxyhemoglobin saturation levels of >90% are considered adequate. Permissive hypercapnia is often well tolerated, provided the pH remains above 7.25.^29^ The normalization of tidal volume to the donor's rather than the recipient's predicted body weight has been demonstrated to reduce the risk of delivering an excessive tidal volume to undersized allografts.^37^, ^38^ To reduce pulmonary vascular resistance and optimize microcirculation, inhaled nitric oxide (iNO) is often used. However, as there is no evidence for efficacy,^39^ routine administration of iNO is not recommended. To minimize barotrauma and to avoid secondary complications such as delirium due to long-term sedation, immobilization, and infections, extubation should be performed as soon as possible.^8^, ^9^, ^15^, ^29^, ^31^, ^32^ In patients with a non-complicated clinical course, cessation of sedation and MV should be completed after 2-3 days.^33^ In cases where weaning proves challenging and MV is necessary for a prolonged period, tracheostomy should be considered to prevent complications associated with prolonged sedation and to facilitate patient mobilization.^40^, ^41^ Non-invasive ventilation is proposed to enable early extubation.^42^ However, controlled studies on the advantages of non-invasive ventilation do not exist. Potential advantages include ensuring adequate oxygenation and decarboxylation, especially when patients are very weak or cannot breathe deeply enough due to pain. In addition, positive end-expiratory pressure may prevent atelectasis. However, some children do not tolerate ventilation masks, or adequate synchronization is not achievable, and insufflation of air into the stomach increases the risk of vomiting and aspiration. High flow nasal cannula is an increasingly used form of respiratory support and oxygen supplementation and often better tolerated.^43^ Early mobilization and chest physiotherapy are very important to facilitate bronchial hygiene and avoid lung atelectasis.^9^, ^29^

### Fluid management

Fluid management exerts a substantial influence on the post-operative course and the emergence of pulmonary and extrapulmonary complications.^9^, ^44^, ^45^ Ischemia and reperfusion injury causes capillary leak, which consequently leads to pulmonary edema. This can be aggravated by several other factors including the use of cardiopulmonary bypass, bleeding complications, blood transfusions, infections, cardiac or renal dysfunction, or hyperinflammation. Therefore, it is imperative to implement a judicious fluid management strategy, which includes the early initiation of diuretics, while concurrently balancing the necessity for volume resuscitation.^8^, ^9^, ^15^, ^17^, ^29^ The initial goal is to maintain adequate end-organ perfusion, which is monitored by measurement of lactate, urine output, and mixed venous oxygen saturation, with the lowest possible cardiac output to reduce the risk of exacerbation of lung edema. If there is evidence of acute kidney injury, early initiation of renal replacement therapy to prevent worsening fluid overload should be considered.^8^, ^46^, ^47^ It is not feasible to formulate a universal recommendation for the intensity of fluid restriction and the application of diuretics; rather, this must be determined on an individual basis, considering graft function, cardiac function, hemodynamic parameters, and kidney function.

## Common perioperative complications

### Primary graft dysfunction

**Figure 2:**
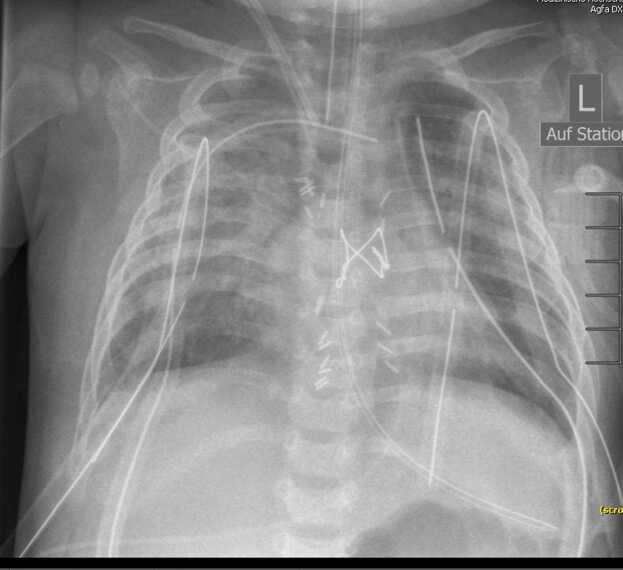
Chest radiograph with ground-glass opacities in a 13-month-old infant with grade 3 PGD after LTX for surfactant protein B mutation.

Primary graft dysfunction (PGD) is the most common complication in the early post-operative period.^13^ PGD is characterized by the presence of pulmonary edema on chest radiographs and impaired pulmonary diffusion capacity (Figure 2). Severity of PGD is graded based on the P/F ratio, which is the partial pressure of arterial oxygen divided by the fraction of inspired oxygen (FiO_2_) (Table 1).^48^ According to the 2016 ISHLT Consensus Group statement, patients should be evaluated for PGD at the reperfusion timepoint after the release of the arterial cross-clamp of the second lung and after 24-, 48-, and 72 hour (T24, T48, and T72).^48^ The incidence of PGD varies in the literature, ranging from 15% to 30%^5^, ^49^ but suggests a prevalence of approximately 30% for all grades and 15% to 20% for grade 3 PGD.^13^ A study of 344 pediatric patients revealed that PGD was the primary indication for the initiation of post-transplant ECMO and the predominant cause of death among patients requiring ECMO.^50^ In addition, studies have demonstrated that PGD is associated with unfavorable short- and long-term outcomes in adults.^5^, ^6^, ^49^ The 30-day mortality rate ranges from 30% to 36%, depending on the severity grade.^13^There are numerous risk factors for the development of PGD (Table 2).^3^, ^13^ Interestingly, in contrast to adults, longer ischemic times have not been shown to adversely affect survival among children who received an allograft for CF.^51^ To minimize the risk of ventilation-induced lung injury and reduce PGD in children, some centers implemented a post-operative extracorporeal life support strategy, particularly when there is evidence of impaired pulmonary gas exchange at T0 or in patients with elevated risk factors, such as PH. There is some evidence that VA-ECMO in the latter group protects the hypoplastic left ventricle by reducing the preload and allows earlier extubation and ambulation compared to those without VA-ECMO.^52^ In patients with severe PGD, the early implementation of extracorporeal life support is beneficial in preventing further ventilator-associated lung injury.^53^ On the other hand, the risk of poor lung perfusion in patients on VA-ECMO, especially in young children on centrally cannulated VA-ECMO, and in addition to the loss of systemic bronchial circulation with transplantation surgery, should be considered and taken into account in the decision for or against ECMO support. Treatment with C1-esterase inhibitors have been shown to shorten the duration of MV in PGD patients but did not have an impact on 1-year survival.^54^ iNO has been demonstrated to improve oxygenation and pulmonary hemodynamics,^55^, ^56^ but its impact on patient long-term outcome remains uncertain. Lung protective ventilation in addition to fluid restriction and the early administration of diuretics, with the simultaneous maintenance of adequate perfusion and the optimization of blood counts is recommended by current guidelines.^13^, ^53^ Re-transplantation (Re-Tx) could be an option in therapy refractory cases; however, Re-Tx in this scenario is associated with a poor outcome, with a 1-year survival of only 39%-54%.^13^, ^57^, ^58^ Therefore, Re-Tx is only justifiable in the absence of concurrent risk factors, including severe infections, cardiac, renal, hepatic dysfunction or neurological complications.

**Table 1:** Definition of Primary Graft Dysfunction (PGD) According to the ISHLT Guidelines 2017

Grade	Pulmonary edema on chest X-ray	PaO_2_/FiO_2_ ratio
PGD grade 0	No	Any
PGD grade 1	Yes	>300
PGD grade 2	Yes	200-300
PGD grade 3	Yes	<200

**Table 2:** Risk Factors for the Development of PGD

• Underlying disease of the recipient and the donor
• Prolonged ischemic time
• Donor-recipient size mismatch
• Intraprocedural complications
• Airway obstruction by secretions or blood
• Arterial or venous anastomotic stenosis
• Left ventricular dysfunction especially in patients who underwent Ltx for PH
• Infections
• Cellular or antibody mediated rejections
• Aspiration
• Lung bleedings
• Transfusion related lung injury
• Hyperinflammation
• Pleural effusions
• Pneumothorax
• Ventilator associated lung injury

### Infections

**Figure 3:**
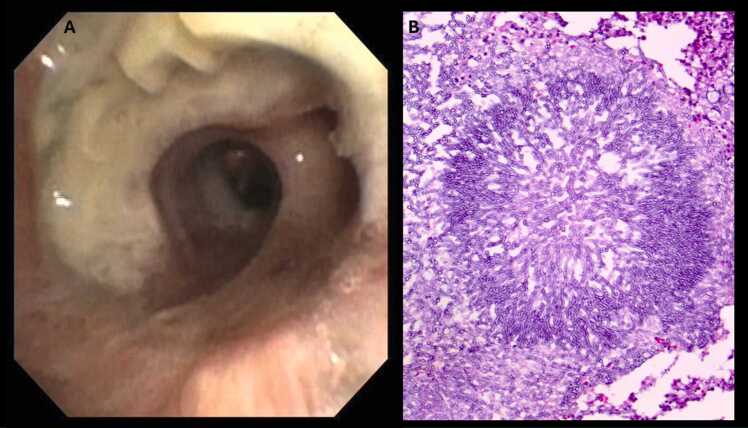
Bronchoscopic view of the right main bronchus in a patient after Re-Tx with newly developed viscous, yellow-gray colored secretions and membranes in the area of the anastomosis 10 days after Ltx **(A)**; histological examination of the extracted membranes showed typical Aspergillus hyphae **(B)**. Of note, this patient was treated with voriconazole and inhaled amphotericin B since Re-Tx.

Infections are a common complication and the main cause of death within the first year after Ltx.^1^ The risk of infection depends on many factors, including the age and thus the immunological competence of the child, the vaccination status, the underlying disease of the recipient, the bacterial colonization of the donor lung, the presence of secretions and atelectasis, the intensity of immunosuppression, the duration of ventilation, the immobilization of the patient, the nutritional status, and compliance with hygienic measures by medical staff.^59^, ^60^, ^61^, ^62^ All patients receive antibiotic treatment during and after transplantation.^8^, ^9^, ^17^, ^61^ In selecting the most appropriate antibiotic, it is imperative to consider the bacterial colonization/infection of the recipient's and donor lungs. There is no evidence that allows a general recommendation regarding the type and duration of antibiotic treatment. However, treatment should not be longer than 14 days if there are no signs of infection. Candida or Aspergillus species can often be isolated from bronchial secretions, especially in patients with CF. This is often a colonization rather than a clinically significant infection. However, Aspergillus species can cause severe infections in the airways and lungs (Figure 3).^63^, ^64^ For this reason, many centers treat all patients prophylactically for a certain period after Ltx.^65^ According to current guidelines, either universal prophylaxis or preemptive therapy can be employed as a strategy to prevent invasive aspergillosis in lung transplant recipients.^66^ Pneumocystis jirovecii can cause severe pneumonia in Ltx recipients. Therefore, prophylaxis with trimethoprim-sulfamethoxazole is usually started after Ltx.^67^ There is no consensus on the appropriate duration of this prophylactic treatment. Many centers have adopted lifelong prophylaxis. Cytomegalovirus (CMV) negative recipients who receive a lung of a CMV positive donor receive CMV-hyperimmunoglobulin after transplantation in addition to antiviral prophylaxis with intravenous ganciclovir followed by oral valganciclovir in many centers, although this prophylactic approach has not been supported by prospective studies. Moreover, CMV and Adenovirus screening, among other viruses, are regulary performed to detect viremia and/or infection at an early stage.

### Neurological complications

There are several neurological complications, and collectively, they affect up to 50% of Ltx recipients and are associated with worse outcomes.^68^ Agitation and delirium are the most common complications. Avoidance of sedation, adequate pain control, and early extubation, as well as parental affection and non-pharmacological interventions, are the most important preventive strategies.^69^ Many patients develop tremors due to calcineurine inhibitors, which many find very uncomfortable. In most cases, however, the tremor diminishes over time or stops completely. Unilateral or bilateral vocal cord paralysis due to laryngeal nerve injury can lead to severe respiratory distress and even the need for MV. Patients often demonstrate stridor and dyspnea, cough, and oxyhemoglobin desaturation during or after feeding. Post-intubation stenosis must be excluded in the differential diagnosis of these symptoms. In addition, following extubation after a period of stability, vocal cord function is assessed to confirm the safety of initiating oral feeding. Horner's syndrome may occur with intraoperative damage to the sympathetic trunk.

Posterior reversible encephalopathy syndrome (PRES) is a serious side effect of calcineurin inhibitors and a potentially life-threatening complication.^70^ Clinical signs include headache, tremor, dizziness, confusion, seizures, depression and coma. If PRES is suspected, magnetic resonance imaging or cranial CT should be performed to prove the diagnosis and to rule out other complications such as hemorrhage, stroke or diffuse cerebral edema. Temporary withholding or reduction of calcineurin inhibitors and substitution with basiliximab or switching from tacrolimus to cyclosporine, with strict therapeutic drug monitoring, may reverse PRES.^70^ Phrenic nerve injury results in impaired ventilation, ventilation perfusion mismatch and may require prolonged MV. A paradoxical breathing pattern during weaning from MV is a typical clinical finding. Diagnosis can be confirmed by sonographic or fluoroscopic assessment of diaphragm function.^71^ Usually, the phrenic nerve recovers over time. In persistent diaphragmatic paralysis, phrenic nerve reconstruction or diaphragm pacing may be a treatment option.^9^, ^72^ Hyperammonemic encephalopathy has been reported in 1%-4% of Ltx recipients.^73^, ^74^ The rapid increase of serum ammonia can cause cerebral edema, coma, and brain death. Therefore, plasma ammonia concentration should be monitored daily for the first week post Ltx and when there is a change in mental status. Lowering protein intake as well as bowel decontamination with lactulose and rifaximin, metronidazole, or neomycin should be considered. Beside arginine and levocarnitine a treatment with ammonia scavengers such as sodium phenylbutyrate, sodium benzoate or sodium phenylacetate as well, as hemodialysis, may be necessary. Currently, there is no consensus of appropriate timing to initiate dialysis, but some clinicians suggest considering dialysis if the ammonia level exceeds three to four times the upper limit of normal.^74^, ^75^ In addition, given the growing body of evidence linking hyperammonemic syndrome to systemic infections with *Ureaplasma* species, prompt initiation of targeted antimicrobial therapy is essential, as hyperammonemia resulting from such infections is considered universally fatal without treatment.^76^ Based on our anecdotal experience, this pathogen appears to occur predominantly in adolescent and adult donors with a history of sexual activity.^77^

### Gastrointestinal complications

Gastrointestinal complications are common following pediatric Ltx. Dysphagia and aspiration are early and potentially, critical concerns. Due to denervation, the cough reflex is often impaired in Ltx recipients, increasing the risk of recurrent aspiration, especially in the presence of vomiting. Gastroparesis leads to impaired gastric emptying, nausea, and vomiting. These gastrointestinal issues can interfere with the absorption of medications, including tacrolimus, leading to fluctuations in immunosuppression levels. Gastroparesis is diagnosed radiologically by gastric emptying and/or barium swallow studies, showing prolonged transit times. Therapeutically, temporary placement of a duodenal feeding tube may be considered, as gastroparesis may resolve over time. In severe cases, injection of botulinum toxin into the pylorus has shown good results in adults.^78^, ^79^ Routine maintenance of a bowel regimen is essential, especially for patients with CF, who are at risk for distal intestinal obstruction syndrome, which requires prompt early evaluation and intervention.^80^ In diarrhea, viral and bacterial infections should be considered, and stool should be analyzed for *Clostridium difficile* toxin, especially if the patient remains on antibiotics. Drug-related gastrointestinal side effects, such as nausea and vomiting (especially due to mycophenolate mofetil) are extremely distressing for children and can lead to a catabolic state and muscle weakness, impairing mobilization. Dimenhydrinate or ondansetron may help to reduce nausea. As opioids can cause impaired intestinal motility, they should only be given with naloxone and only for the time that is necessary. Preventive strategies, including the use of proton pump inhibitors or histamine antagonists, are standard in most centers to reduce gastroesophageal reflux, which occurs in up to 90% of pediatric Ltx recipients^81^ and is a risk factor for recurrent aspiration and chronic lung allograft dysfunction.^82^ In severe cases, surgical fundoplication should be considered, but as it may lead to further complications, its indication should be carefully evaluated through interdisciplinary assessment.^78^, ^79^, ^80^, ^81^, ^82^

### Renal complications

Acute kidney injury and chronic kidney failure occur often after Ltx in children, with incidence ranging from 40% to 75% and is associated with significant morbidity and mortality. Risk factors for renal dysfunction in general include young age, pre-transplant renal dysfunction, pre- and post-transplant ECMO support, excessive transfusions, nephrotoxic drugs such as calcineurin inhibitors, and certain antibiotics such as aminoglycosides or vancomycin.^83^, ^84^. Notably, the need for dialysis in children after Ltx had significantly worse outcomes and was associated with lower estimated glomerular filtration rate at transplant.^85^

Although fluid restriction is recommended to prevent pulmonary edema, adequate organ perfusion should always be ensured to avoid prerenal renal failure, and nephrotoxic drugs should be avoided as much as possible.^9^, ^29^ Regarding calcineurin inhibitors, this could be achieved, for example, by induction therapy, which may allow delayed administration or acceptance of lower plasma levels of tacrolimus in the first days after transplantation.^86^ In addition to regular fluid balance, creatinine, urea, and glomerular filtration rate should be measured daily to detect the onset of renal failure. In this case, hemodialysis should be considered at an early stage.^47^

### Atrial arrythmias

Atrial arrythmias like atrial fibrillation, atrial tachycardia, or atrial flutter are relatively common after Ltx affecting about 15%-20% of pediatric Ltx recipients.^87^, ^88^, ^89^ Contributing factors are left atrial incisions and suture lines during transplantation, electrolyte imbalances, pain, and stress. Atrial arrythmias have been reported to eventually increase mortality and hospital stay in adults. Although data on atrial arrythmias in children following Ltx are scarce,^87^, ^89^ this has been reported in teens > 12 years of age but did not have a negative effect on hospital length of stay or early mortality in these cases.^90^ Rate control with a beta-blocker is offered when hemodynamically stable, and synchronized cardioversion is performed when rate control alone is insufficient. For those with recurrent episodes, amiodarone or beta-blocker therapy is usually initiated.^87^, ^89^

## Conclusion

Post-operative care for children after Ltx is challenging and requires vigilance to reduce lung allograft injury and to minimize complications in other organ systems. Optimal intensive care unit management for these complex patients is imperative for successful outcomes for pediatric Ltx recipients.

## Declaration of Competing Interest

The authors declare that they have no known competing financial interests or personal relationships that could have appeared to influence the work reported in this paper.
